# Polychlorinated Biphenyls Alter Estrogen Receptor β-mediated Epigenetic Regulation, Promoting Endometriosis

**DOI:** 10.1210/endocr/bqaf146

**Published:** 2025-10-07

**Authors:** Yuri Park, Nuri Sung, Eunsu Kim, Jaeyeong Jeong, Juhee Sim, Mi Jin Park, John P Lydon, Xiaoming Guan, Sang Jun Han

**Affiliations:** Department of Molecular and Cellular Biology, Baylor College of Medicine, Houston, TX 77030, USA; Department of Molecular and Cellular Biology, Baylor College of Medicine, Houston, TX 77030, USA; Department of Molecular and Cellular Biology, Baylor College of Medicine, Houston, TX 77030, USA; Department of Molecular and Cellular Biology, Baylor College of Medicine, Houston, TX 77030, USA; Department of Molecular and Cellular Biology, Baylor College of Medicine, Houston, TX 77030, USA; Department of Molecular and Cellular Biology, Baylor College of Medicine, Houston, TX 77030, USA; Department of Molecular and Cellular Biology, Baylor College of Medicine, Houston, TX 77030, USA; Department of Obstetrics and Gynecology, Baylor College of Medicine, Houston, TX 77030, USA; Department of Molecular and Cellular Biology, Baylor College of Medicine, Houston, TX 77030, USA

**Keywords:** PCB126, endometriosis, ESR2, DNMT3A, AXL, GAS6

## Abstract

Endometriosis is a pathological condition characterized by the ectopic growth of endometrial cells, leading to chronic pelvic pain and infertility. Epidemiological studies have associated exposure to dioxin-like polychlorinated biphenyls, particularly PCB126, with an increased risk of endometriosis. However, the underlying mechanisms of this association remain poorly understood. We utilized a surgically induced endometriosis mouse model and human endometrial cell lines to assess the impact of PCB126 on endometriosis progression. Mice were exposed to environmentally relevant doses of PCB126. Endometriotic lesion growth, estrogen receptor signaling, receptor tyrosine kinase activity, and gene expression changes induced by PCB126-mediated elevation of DNA methyltransferase 3A (DNMT3A) were evaluated using histology, bioluminescent imaging, immunoblotting, and RNA sequencing. Functional validation was conducted using a pharmacologic AXL inhibitor and tissue-specific *Dnmt3a* knockout mice. PCB126 significantly promoted the growth of ectopic lesions and humanized models of endometriosis. Mechanistically, PCB126 enhanced estrogen receptor β (ESR2) activity by upregulating AXL and its ligand, growth arrest–specific 6, and elevating DNMT3A expression. The inhibition of AXL signaling suppressed the growth of endometriotic lesions. ESR2 directly regulated *Dnmt3a* expression, and loss of *Dnmt3a* reduced lesion growth and inflammatory cytokine production, thereby reversing immune dysregulation. These findings establish a mechanistic link between PCB126 exposure and epigenetic and immune reprogramming in endometriotic lesions. Our findings establish a mechanistic connection between environmental PCB126 exposure and endometriosis progression via the AXL/ESR2/DNMT3A axis. This study provides new insight into how endocrine-disrupting chemicals promote hormone-sensitive diseases through epigenetic and immunological pathways, offering potential targets for therapeutic intervention.

Endometriosis is a medical condition in which endometrial cells grow outside the uterine cavity, affecting up to 10% of women of reproductive age and causing symptoms such as infertility and severe pelvic pain (1). Due to the severe chronic morbidities associated with endometriosis, numerous studies have sought to identify the distinct molecular features of endometriotic lesions to develop more effective prognostic, diagnostic, and therapeutic strategies.

Several hypotheses have been proposed to explain the initiation and progression of endometriosis in approximately 10% of women of reproductive age (2). Among these, exposure to environmental endocrine disruptors—such as polychlorinated biphenyls (PCBs), dioxins, bisphenol A, and phthalates—has been identified as a potential contributing factor in the progression of endometriosis (3). For example, women exposed to moderate levels of PCBs [5-8 parts per billion (ppb)] or high levels (>8 ppb) have a higher incidence of endometriosis compared to those with low PCB exposure (<5 ppb) (4). In addition, elevated serum levels of dioxin-like PCB congeners have been detected in Italian women with endometriosis compared to healthy controls (5). A study conducted in Belgium, Italy, and India found elevated total PCB concentrations in patients with adenomyosis and peritoneal endometriosis compared to controls (6). The primary route of human exposure to PCBs is through the consumption of contaminated food and occupational/industrial waste accidents (7-9). Thus, accumulating evidence supports the hypothesis that women with high levels of PCB exposure are at increased risk of developing endometriosis compared to those with lower exposure levels.

Among PCBs, we are particularly interested in 3, 3′, 4, 4′, 5-pentachlorobiphenyl (PCB126) due to its unique molecular properties associated with endometriosis. PCB126 is considered the most potent dioxin-like PCB and has the highest toxic equivalency factor among PCBs, at 0.1 second only to TCDD, which has a toxic equivalency factor of 1.0. Due to its high potency, PCB126 is often the primary contributor to the toxic equivalency in common PCB mixtures, accounting for up to 90% of the total dioxin-like activity (10). Elevated levels of PCB126 have been detected in the adipose tissue of patients with deep-infiltrating endometriosis (8). PCB126 exposure stimulates the mRNA expression of steroidogenic genes, such as steroidogenic acute regulatory protein, hydroxy-delta-5-steroid dehydrogenase 3 β, and cytochrome P450 family 19 subfamily A member 1, in chicken ovarian follicles, leading to elevated local estrogen levels, a key feature of endometriosis (11, 12).

PCB126 exhibits estrogenic activity in certain contexts, although its effects are complex. For example, PCB126 enhances estradiol (E_2_) production in fish ovarian follicles, mimicking the effects of exogenous E_2_ by stimulating aromatase activity and modulating steroidogenic enzyme pathways (13). Additionally, PCB126 induces estrogen receptor (ER)-responsive genes alongside aryl hydrocarbon receptor (AhR)-mediated responses (14). This study revealed that PCB126 may promote estrogen-dependent disease progression, such as endometriosis, by facilitating crosstalk between AhR signaling and ER pathways.

PCB126 exposure induces dynamic changes in epigenetic regulation, including DNA methylation, histone modifications, and RNA methylation, in a tissue-dependent manner. Prenatal exposure to PCB126 in mice upregulates Dnmt1 and Dnmt3b expression in the fetal liver, resulting in global DNA hypermethylation (15). Exposure to PCB126 also upregulates histone lysine methyltransferases, such as kmt2a and setd1a, in zebrafish, leading to altered chromatin accessibility (16). Alterations in epigenetic regulation are significantly associated with endometriosis progression. For example, dysregulated expression of DNA methyltransferases (DNMT) [DNA methyltransferase 1 (DNMT1), DNA methyltransferase 3A (DNMT3A), and DNA methyltransferase 3B (DNMT3B)] in endometriotic lesions, along with differential DNA methylation patterns in genes involved in inflammatory pathways, steroid signaling, and apoptosis, have been linked to the progression of endometriosis (17, 18).

These observations strongly suggest that exposure to PCB126 may be a causal factor in the initiation and progression of endometriosis. However, the precise mechanisms by which PCB126 promotes endometriosis progression remain unclear.

## Material and Methods

### Rigorous Experimental Design

All investigators were blinded to mouse genotype information. All in vitro and animal experiments were independently repeated 3 times to validate the results.

### Animal Numbers and Power Calculations

The required minimal number of animals per group was determined using a power calculation to ensure adequate statistical power (α = .05, power = 80%) to detect a biologically significant difference. Sample size estimation was performed using GPower (19) based on the data from each experiment.

### Mice

C57BL/6J female mice (6 weeks old) and severe combined immunodeficiency (SCID) female mice (6 weeks old) were purchased from Jackson Laboratory. C57BL/6J, SCID, ROSA^LSL:ESR2/+^ :progesterone receptor (PR)^Cre/+^ (20) and PR^Cre/+^ (21), Dnmt3a^f/f^ (22), and Dnmt3a^f/f^:PR^Cre/+^ mice were maintained in the designated animal care facility at Baylor College of Medicine according to the Institutional Animal Care and Use Committee guidelines for the care and use of laboratory animals. An Institutional Animal Care and Use Committee-approved protocol was followed for all animal experiments in this study. The assurance number of our animal protocol is D16-00475.

### Immortalized Human Endometrial Cells

Immortalized human endometrial stromal cells (IHESCs) (23) and EMosis-CC/TERT1 [immortalized human endometriotic epithelial cells (IHEECs)] (24), enhanced estrogen receptor β (ESR2) overexpressing immortalized human endometrial epithelial cells (IHEESCs) (20) were employed and confirmed by short tandem repeat profiling; these cells were not contaminated with mycoplasma.

### PCB126 Effect on Steroid Receptor Coactivator-1 Isoform, ESR2, and Matrix Metalloproteinase-9 in IHEECs

For the vitro experiment to define the role of PCB126, IHEECs and IHESCs were cultured in DMEM/F12 supplemented with 10% fetal bovine serum (FBS), as these cells require estrogen for optimal growth. IHEECs were treated with 0.1 nM PCB126 or vehicle control for 24 hours in DMEM/F12 medium supplemented with 10% FBS, penicillin (100 U/mL), streptomycin (100 µg/mL), and amphotericin B (2.5 µg/mL) under humidified conditions (5% CO_2_, 95% air) at 37 °C. Following treatment, total protein lysates were prepared from PCB126- and vehicle-treated IHEECs. The levels of steroid receptor coactivator-1 (SRC-1) isoform, ESR2, and matrix metalloproteinase-9 (MMP9) were assessed by Western blot analysis. Tubulin was used as a loading control.

### 3-(4,5-Dimethylthiazol-2-yl)-5-(3-carboxymethoxyphenyl)-2-(4-sulfophenyl)-2H-tetrazolium) Growth Assay

Human endometrial cells were seeded into 96-well plates at a density of 1 × 10^4^ cells per well. The following day, cells were treated with serial dilutions of PCB126. After 3 days of treatment, 10μL of 3-(4,5-dimethylthiazol-2-yl)-5-(3-carboxymethoxyphenyl)-2-(4-sulfophenyl)-2H-tetrazolium (MTS) reagent (Promega, catalog number: G1111) was added to each well, and the plates were incubated for 2 hours. Optical density was then measured at 450 nm using a microplate reader.

### Surgically Induced Endometriosis (Autotransplantation)

Endometriosis was surgically induced in mice under anesthesia and aseptic conditions using a modified method described previously (25). Briefly, C57BL/6J female mice (6 weeks old) were implanted subcutaneously with a sterile 60-day release pellet containing 0.36 mg of 17-β estradiol (Innovative Research of America, Sarasota, FL, USA). Two days after the implantation, 1 uterine horn from each mouse was isolated under anesthesia. In a Petri dish containing prewarmed DMEM/F-12 medium (Invitrogen) supplemented with 100 U/mL penicillin and 100 µg/mL streptomycin, the isolated uterine horns were longitudinally incised with scissors to expose the endometrium. A 2-mm endometrial fragment was then excised using a dermal biopsy punch (Miltex) and sutured to the mesenteric membrane attached to the intestine of the same mouse through a midline abdominal incision, using a 7-0 braided polypropylene suture (Ethicon). For endometriosis control mice, the suture was performed without endometrial tissue implantation. The abdominal incision was closed using a 5-0 braided polypropylene suture (Ethicon) in a continuous manner. On day 21 postsurgery, mice were euthanized, and both endometriotic lesions and eutopic endometria were carefully dissected from the surrounding tissues. In control mice, the uterus was collected 21 days after the sham endometriosis procedure, which involved making an abdominal incision and then closing it without implanting endometrial fragments. The volume of endometriotic lesions was calculated using the formula: volume (mm^3^) = 0.52 × width × length × height.

### Endometriosis by Heterotransplantation With Cultured Human Endometrial Cells

For noninvasive analysis of ectopic lesion growth in SCID mice, luciferase-labeled IHEECs and luciferase-labeled IHESCs were used (20). Cells were cultured in DMEM/F12 medium supplemented with 10% FBS, penicillin (100 U/mL), streptomycin (100 µg/mL), and amphotericin B (2.5 µg/mL) under humidified conditions (5% CO_2_, 95% air) at 37 °C. The culture medium was replaced every other day. On the day of transplantation, cells were trypsinized using 0.1% trypsin-EDTA. Luciferase-labeled IHESCs (2 × 10^6^ cells) were mixed with luciferase-labeled IHEECs (2 × 10^6^ cells) in 10 mL of DMEM/F12, pelleted, and washed. The mixed cell pellet was resuspended in 100 µL of DMEM/F12 and combined with 100 µL of Matrigel (BD Biosciences) at a 1 : 1 ratio. A total of 200 µL of the cell-Matrigel suspension was injected intraperitoneally along the midventral line just caudal to the umbilicus of SCID female mice (6 weeks old) previously implanted with a sterile 60-day release pellet containing 0.36 mg of 17-β estradiol (Innovative Research of America).

### Endometriosis to Generate Dnmt3a Knockout Ectopic Lesions

Dnmt3^f/f^:PR^Cre/+^ and Dnmt3a^f/f^ female mice (n = 4 per group, 8 weeks old) were subcutaneously injected with 17β-E_2_ at a dose of 100 ng per 20 g of body weight once daily for 3 consecutive days. After treatment, uterine horns from each mouse were isolated under anesthesia and longitudinally incised with scissors. A 2-mm endometrial fragment was then obtained using a dermal biopsy punch. Syngeneic recipient C57BL/6J female mice (8 weeks old, n = 8) were implanted subcutaneously with a sterile, 60-day release pellet containing 0.36 mg of 17β-estradiol (Innovative Research of America) 2 days before endometriosis surgery. On the day of surgery, 1 endometrial fragment from a donor mouse was sutured to the mesenteric membrane of a recipient mouse through a midline abdominal incision using a 7-0 braided polypropylene suture (Ethicon). The incision was then closed with a 5-0 braided polypropylene suture (Ethicon) in a continuous pattern. On day 21 following endometriosis induction, mice were euthanized, and endometriotic lesions as well as eutopic endometria were carefully dissected from surrounding tissues. Lesion volume was calculated using the formula: volume (mm^3^) = 0.52 × width × length × height. Although the minimum number of animals required for endometriosis methods to assess the effect of Dnmt3a knockout (KO) on endometriosis, achieving 80% power with a *P*-value of .05, is 1 mouse, each group in this study included 4 mice to improve statistical robustness.

### PCB126 Exposure During Endometriosis Progression

After endometriosis was induced in mice using both auto- and heterotransplantation methods, the mice were randomly divided into 2 groups. One group received intraperitoneal injections of PCB126 at a dose of 1 mg/kg once per week for 5 weeks. The control group received intraperitoneal injections of vehicles alone on the same schedule. The minimum number of animals required for both auto- and heterotransplantation methods to assess the effect of PCB126 on endometriosis, achieving 80% power with a *P*-value of .05, is 2 mice.

### BMS-777607 Inhibits Endometriosis Progression

After endometriosis was induced in mice using autotransplantation, the mice were randomly divided into 2 groups. One group received intraperitoneal injections of BMS-777607 (Axl inhibitor, 25 mg/kg; Selleckchem, catalog number: S1562) 5 times per week for 3 weeks. The control group received intraperitoneal injections of vehicles (5% DMSO) on the same schedule. One mouse is the minimum number of animals required for both autotransplantation methods to assess the effect of BMS-777607, Axl inhibitor, on endometriosis, achieving 90% power with a *P*-value of .01.

### Mouse Receptor Tyrosine Kinase Array Analysis

To assess differential receptor tyrosine kinase (RTK) activation between normal and endometriotic tissues, as well as between ectopic lesions exposed to PCB126 or vehicle, the Mouse Phospho-RTK Array Kit (R&D Systems, catalog number: ARY014) was used according to the manufacturer's instructions. Briefly, control mice with sham endometriosis and mice with endometriosis were treated with PCB126 or vehicle (5% DMSO) as described previously. Subsequently, normal uterine tissue from control mice, as well as ectopic lesions and eutopic endometrium from endometriosis mice, was freshly harvested. Total cell lysates were prepared from the isolated tissues. Equal amounts of protein (300 µg per array membrane) from each sample group were incubated with preblocked phospho-RTK array membranes overnight at 4 °C on a rocking platform. After washing to remove unbound proteins, the membranes were incubated with a cocktail of biotinylated antiphosphotyrosine detection antibodies, followed by a streptavidin-horseradish peroxidase conjugate. Chemiluminescent signals were developed using the supplied substrate and visualized with a chemiluminescent imaging system. Dot intensities corresponding to phosphorylated RTKs were quantified using ImageJ software, normalized to internal reference spots, and compared across sample groups to identify differentially activated RTKs.

### Western Blot Analyses

Primary antibodies against the following proteins were used: SRC-1 (RRID:AB_297046, 1:1000 dilution), ESR2 antibody (RRID:AB_1964229, 1:1000 dilution), MMP9 (RRID:AB_776512, 1:1000 dilution), Dnmt1(RRID:AB_10710384, 1:1000 dilution), Dnmt3a (RRID:AB_10844010, 1:1000 dilution), Dnmt3b (RRID:AB_2094130, 1:1000 dilution), and α-tubulin (RRID:AB_2210548, 1:5000 dilution). Membranes were incubated with horseradish peroxidase-conjugated secondary antibodies (RRID:AB_330924, 1:5000 dilution or RRID:AB_2099233, 1:5000 dilution), and signals were detected using the SuperSignal™ West Femto Chemiluminescent Substrate (Thermo Fisher Scientific).

### Imaging and Quantification of Bioluminescence Data

Mice were anesthetized with 1.5% isoflurane in air using an inhalation anesthesia system (VetEquip). D-Luciferin (Xenogen) was administered via intraperitoneal injection at a dose of 40 mg/kg body weight. Ten minutes after injection, mice were imaged using an IVIS Imaging System (Xenogen) under continuous isoflurane anesthesia (1–2%). Imaging parameters were kept consistent across all sessions for comparative analysis. Grayscale reference images were superimposed with pseudocolor images representing bioluminescence signals and analyzed using Living Image software (Version 4.4, Xenogen). A region of interest (ROI) was manually drawn over the area of signal intensity, and the ROI size was kept constant across all samples. Signal intensity was quantified as total photon flux (photons/sec/cm^2^) within the ROI.

### PCB126 Effect on Estrogen Receptor α and ESR2 Activity

HeLa cells were cultured in phenol red–free DMEM supplemented with 10% charcoal-stripped serum. Cells were then plated into 24-well plates. Once they reached approximately 90% confluence, HeLa cells were transiently transfected using Lipofectamine 2000 (Thermo Fisher Scientific, catalog number: [11668019]) with an ESR2 expression vector in the pCR3.1 backbone (5 ng/well) and a luciferase reporter plasmid containing 5 copies of the estrogen response element (ERE; 400 ng/well). As a control for ESR2, a parallel group of HeLa cells was transfected with an estrogen receptor α (ESR1) expression vector in the same vector backbone (5 ng/well) along with the same ERE-luciferase reporter (400 ng/well). Two days posttransfection, cells were treated with PCB126 at concentrations ranging from 0 to 200 nM for an additional 2 days. As a positive control, a separate set of cells was treated with 10 nM estradiol for 2 days. Luciferase activity was measured using the Luciferase Assay System (Promega, catalog number: E1500) according to the manufacturer's instructions.

### PCB126 Effects on GAS6 and ESR2 Expression Levels Using RT-qPCR

IHEECs and IHESCs were cultured as previously described. To assess whether PCB126 increases GAS6 and ESR2 expression levels, cells were seeded in 6-well plates 1 day before treatment. Once the cells reached approximately 80% confluency, various concentrations of PCB126, diluted in DMSO, were added. After 24 hours of treatment, the total RNA was isolated using the RNeasy Plus Mini Kit (Qiagen, catalog number: 74134). First-strand cDNA was synthesized from 1 µg of total RNA using the SuperScript II Reverse Transcriptase Kit (Invitrogen, catalog number: 18064022) according to the manufacturer's instructions. Gene expression levels of GAS6 and ESR2 were quantified using TaqMan probes for GAS6 (Invitrogen, catalog number: Hs01090305_m1) and ESR2 (Invitrogen, catalog number: Hs00230957_m1). Relative mRNA expression was calculated using the 2^−ΔΔCT^ method and normalized to 18S rRNA levels.

### Effect of GAS6 on ESR2 Transcriptional Activity Using the ERE Luciferase Assay

HeLa cells were seeded in 24-well plates and cultured in phenol red–free DMEM supplemented with 10% charcoal-stripped serum. When cells reached approximately 80% confluency, they were transiently transfected with 500 ng of the pCR3.1-ERE-luc reporter plasmid and 50 ng of the ESR2 expression plasmid (pCR3.1-ESR2) using Lipofectamine 2000 (Thermo Fisher Scientific, catalog number: 116680300), following the manufacturer's protocol. Three days after transfection, cells were treated with varying concentrations (0-100 ng/mL) of GAS6 protein (R&D Systems, catalog number: 885-GSB). After 48 hours of treatment, relative luciferase activity was measured using the Luciferase Assay System (Promega, catalog number: E1500).

### RNA Sequencing Analysis of Dnmt3a Ectopic Lesions

Dnmt3a KO ectopic lesions (n = 3) and wild-type (WT) control lesions (n = 3) were generated using Dnmt3a^f/f^ :PR^Cre/+^ (8 weeks old, n = 3) and Dnmt3a^f/f^ (8 weeks old, n = 3) female donor mice, as described earlier. Total RNA was extracted from Dnmt3a KO and control ectopic lesions using the RNeasy Plus Mini Kit (Qiagen, catalog number: 74134), according to the manufacturer's instructions. To minimize genomic DNA contamination, the spin column membrane was additionally treated with DNase I (2 U/μL). RNA quality was assessed using a NanoDrop spectrophotometer, Invitrogen Qubit 2.0 fluorometric quantitation assay, and Agilent Bioanalyzer. RNA libraries were prepared using the Illumina TruSeq Stranded mRNA Library Preparation Kit. Sequence reads were trimmed to remove adapter sequences and low-quality bases using Galaxy version 23.1.rc1 (26). Trimmed sequence reads were mapped to the GRCm38 reference genome. Read count extraction and normalization were subsequently performed using tools on the Galaxy platform. The processed data was visualized as a heatmap using the heatmap generation tool in Galaxy version 23.1.rc1.

### Formalin-fixed Paraffin-embedded for Human Endometriotic Lesions and Normal Endometrium

Ovarian endometriomas were obtained from patients with endometriosis during surgical procedures at Baylor College of Medicine under an institutional review board-approved protocol. Normal endometrial tissue was collected from uteri removed during hysterectomies performed for uterine fibroids, also under an institutional review board-approved protocol at Baylor College of Medicine. All patients had discontinued exogenous hormonal treatments for at least 3 months prior to surgery. Both ovarian endometriomas and normal endometrial tissues were fixed in 10% buffered formalin phosphate for 24 hours and subsequently stored in 70% ethanol for the formalin-fixed paraffin-embedded processing. The tissues were dehydrated using ethanol and xylene in a tissue processor and then embedded in paraffin.

### Immunohistochemistry

The paraffin-embedded tissues were sectioned at a thickness of 7 μm. The sections were deparaffinized in xylene, rehydrated through a graded ethanol series, and then subjected to immunostaining. Antigen retrieval was performed using a citrate-based buffer (pH 6.0; Vector, catalog number: H-3300). Antibodies against Dnmt3a (RRID:AB_10844010, 1:300 dilution) and Ki67 (RRID:AB_302459, 1:1000 dilution) were used. Specific antigens were visualized using a DAB substrate kit (Vector, catalog number: SK-4100). H-scores of the stained images were quantified using QuPath software.

### Gene Expression Omnibus

RNA expression profiles comparing control and Dnmt3a KO ectopic lesions in mice with endometriosis have been deposited in the Gene Expression Omnibus database under accession number GSE296259.

### Statistical Analysis

An independent 2-tailed Student's *t*-test was applied for the 2-group comparison. In the case of multiple comparisons, a 1-way ANOVA with a post hoc Tukey test was applied. All statistical analyses were performed using GraphPad Prism version 8.0. A *P*-value of <.05 was considered statistically significant.

## Results

### The PCB126 Exposure Elevates Endometriosis Progression

**Figure 1.**
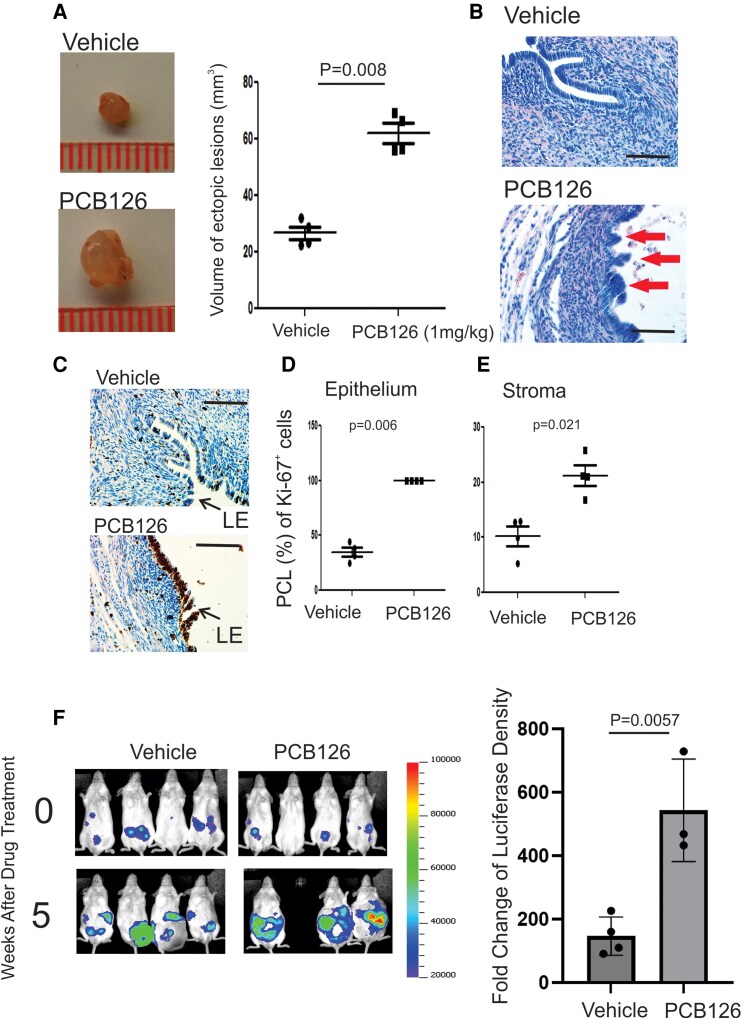
PCB126-enhanced endometriosis progression in mice with endometriosis. (A) Volume analysis of ectopic lesions isolated from mice with endometriosis treated with PCB126 (1 mg/kg) or vehicle, administered once weekly for 5 weeks (n = 4 per group). (B) Hematoxylin and eosin staining of ectopic lesions from vehicle- and PCB126-treated mice shown in (A). The red arrowhead indicates the atypical endometriotic lesions. (C) Immunohistochemical analysis of Ki-67 expression in ectopic lesions treated with vehicle or PCB126. (D, E) Quantification of the percentage of Ki-67–positive cells in the epithelial (D) and stromal (E) compartments of ectopic lesions from vehicle- and PCB126-treated groups by using QuPath program (n = 4 per group) (28). (F) Evaluation of PCB126-induced progression of human ectopic lesions in SCID mice. Luciferase activity was imaged using in vivo imaging systems before and after 5 weeks of treatment with PCB126 (1 mg/kg) or vehicle. Luciferase fold change was calculated as the ratio of post- to pretreatment signal. One mouse with endometriosis treated with PCB126 died after 3 weeks of treatment. Immunohistochemistry scale bar, 50 μm.

Abbreviations: LE, luminal epithelium; SCID, severe combined immunodeficiency.

Elevated levels of PCB126 are correlated with the progression of endometriosis in women (27). However, there is no direct evidence demonstrating whether and how PCB126 exposure promotes the progression of endometriosis. To address this critical question, endometriosis was surgically induced in female mice using the autotransplantation method, followed by intraperitoneal injection of PCB126 (1 mg/kg) or vehicle as a control. Compared to vehicle-treated mice, PCB126 exposure significantly increased the volume of ectopic lesions (Fig. 1A). In addition to lesion enlargement, hematoxylin and eosin staining revealed that PCB126 induced multilayered epithelial structures within the endometriotic lesions, a feature not observed in the vehicle-treated group (Fig. 1B). Furthermore, immunohistochemistry (IHC) for Ki-67, a proliferation marker, showed significantly increased cellular proliferation in both epithelial and stromal compartments of the lesions in PCB126-treated mice compared to controls (Fig. 1C, 1D, and 1E). These findings indicate that PCB126 exposure enhances the proliferation of epithelial and stromal cells, promoting an atypical and more aggressive form of endometriosis in this murine model.

These observations also raised the question of whether PCB126 exposure promotes the progression of human endometriosis. To investigate this, we injected a mixture of IHEECs and IHESCs into SCID mice to generate human endometriotic lesions using a heterotransplantation method, as described in a previous study (20). To noninvasively monitor the growth of human endometriotic lesions in mice, luciferase-labeled immortalized human endometrial epithelial and stromal cells were used (20). Comparative analysis of in vivo luciferase imaging revealed that PCB126 exposure significantly increased luciferase activity in human endometriotic lesions in SCID mice compared to vehicle-treated controls (Fig. 1F). These findings indicate that PCB126 exposure promotes the progression of both murine and human endometriotic lesions in mouse models.

### The Exposure of PCB126 Elevated the Endometriosis Driver in Endometriotic Lesions

**Figure 2.**
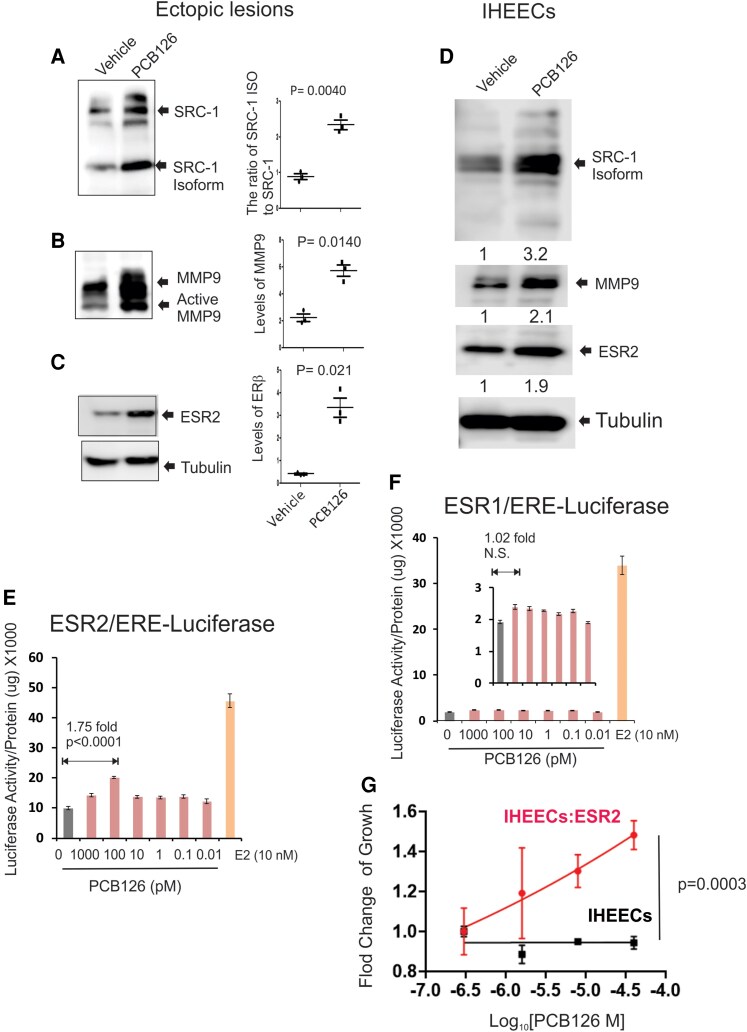
PCB126 elevated the SRC-1 isoform/MMP9/ERβ axis in endometriotic lesions. (A-C) Western blot analysis to assess levels of SRC-1 and its isoform (A), MMP9 (B), and ESR2 (C) in ectopic lesions isolated from mice with endometriosis treated with PCB126 (1 mg/kg) or vehicle once weekly for 5 weeks. Tubulin was used as a loading control for normalization. (D) Western blot analysis of SRC-1 isoform, MMP9, and ESR2 in IHEECs treated with 0.1 nM PCB126 or vehicle for 2 days. Tubulin was used for normalization. (E, F) Luciferase reporter assay to evaluate the effect of PCB126 on the intrinsic transcriptional activity of ESR2 (E) and ESR1 (F) in HeLa cells. Cells were transiently transfected with ESR2 or ESR1 expression vectors and an ERE-luciferase reporter construct, followed by treatment with varying doses of PCB126 or vehicle. As a positive control for ER activation, cells were treated with 10 nM estradiol. (G) Assessment of cell proliferation in IHEECs expressing ESR2 vs parental IHEECs after treatment with increasing concentrations of PCB126 for 3 days. Cell growth was measured using the MTS assay, and fold change was calculated as the ratio of cell growth under each treatment condition relative to the vehicle control. Abbreviations: ER, estrogen receptor; ERE, estrogen response element; ESR2, estrogen receptor β; IHEEC, immortalized human endometrial epithelial cell; MMP9, matrix metalloproteinase-9; MTS, 3-(4,5-dimethylthiazol-2-yl)-5-(3-carboxymethoxyphenyl)-2-(4-sulfophenyl)-2H-tetrazolium; SRC-1, steroid receptor coactivator-1.

The SRC-1 isoform/MMP-9/ESR2 axis has been identified as a key driver of endometriosis by inhibiting apoptosis and promoting inflammatory responses within endometriotic lesions (20, 25). Thus, we investigated whether PCB126 exposure modulates the SRC-1 isoform/MMP-9/ESR2 axis in endometriotic lesions, enhancing endometriosis progression. Endometriotic lesions were isolated from mice with endometriosis treated with PCB126 or vehicle control, and the levels of this regulatory axis were analyzed by Western blot. PCB126 exposure significantly increased the ratio of the SRC-1 isoform to full-length SRC-1 in endometriotic lesions compared to vehicle-treated controls (Fig. 2A). In addition, PCB126 elevated the protein levels of MMP-9 and ESR2 in endometriotic lesions (Fig. 2B and 2C). These results indicate that PCB126 enhances the SRC-1 isoform/MMP-9/ESR2 axis, contributing to the progression of endometriosis in this mouse model. To further validate whether PCB126 exposure also activates this axis in human endometriotic lesions, we employed IHEECs derived from ovarian endometrioma patients (29). PCB126 exposure (0.1 nM) increased the levels of the SRC-1 isoform, MMP-9, and ESR2 in IHEECs compared to vehicle-treated controls, consistent with observations in mouse endometriotic lesions (Fig. 2D). The 0.1 nM PCB126 is biologically relevant in vitro subnanomolar range (∼0.01-1 nM) of PCB126 is widely regarded as biologically active in human in vitro systems (30). These findings indicate that PCB126 enhances the SRC-1 isoform/MMP-9/ESR2 axis in both mouse and human endometriotic lesions. Endometriosis is an estrogen-dependent disease, with both ESR2 and ESR1 playing critical roles in its progression (31, 32). However, ERS2 levels were markedly elevated in endometriotic lesions compared to ESR1 (33). Therefore, whether PCB126 activates both ERs or selectively targets 1 subtype remains unclear. To address this key question, HeLa cells were used, as they are widely regarded as ESR1- and ESR2-negative and thus serve as an ideal model system for studying exogenous ER activation (34). To assess ER activity, HeLa cells were transiently transfected with either an ESR1 or ESR2 expression vector along with a luciferase reporter containing an ERE. As a control, we evaluated the effect of estradiol (10 nM) on ER activity in HeLa cells. Estradiol significantly increased the transcriptional activity of both ESR1 and ESR2 compared to vehicle-treated cells (Fig. 2E and 2F). Using this system, we next assessed whether PCB126 activates ERs. PCB126 (0.1 nM) significantly increased ESR2 transcriptional activity compared to the vehicle (Fig. 2E), whereas it did not induce ESR1 activity (Fig. 2F). These results indicate that a lower dose of PCB126 increases ESR2, but not ESR1, activity compared to the vehicle.

To validate whether the PCB126/ESR2 axis drives endometriosis progression, we employed IHEECs overexpressing ESR2 (IHEECs:ESR2) (20) and then determined whether PCB126 enhances the growth of IHEECs:ER2 compared to their parental IHEECs. The MTS cell proliferation assay revealed that PCB126 significantly increased the proliferation of IHEECs:ESR2 compared to control IHEECs (Fig. 2G). These results suggest that PCB126 promotes the proliferation of IHEECs primarily through ESR2 activation.

### PCB126 Activates the AXL/GAS6 Axis to Enhance ERβ Activity in Endometriotic Lesions

**Figure 3.**
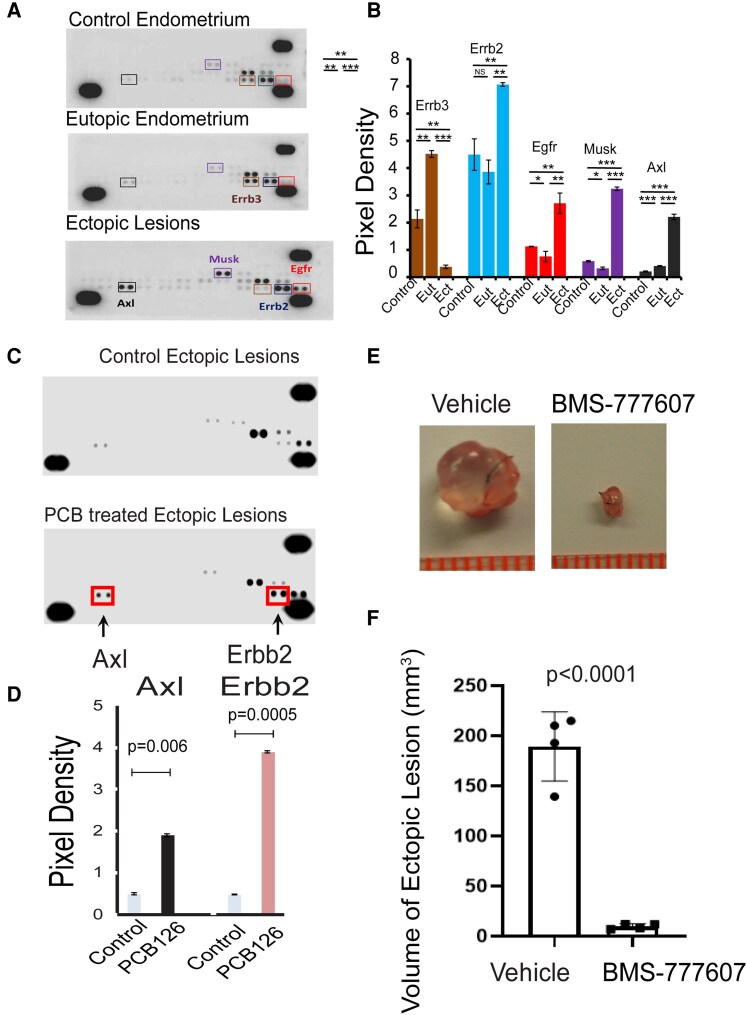
PCB126 activates AXL signaling to enhance endometriosis progression. (A, B) Analysis of RTK activation in ectopic lesions and eutopic endometrium of mice with endometriosis, as well as control endometrium from nonendometriosis control mice (n = 3 per group). Phosphorylated RTKs were detected using the Mouse Phospho-Receptor Tyrosine Kinase Array Kit (A). Differential levels of phosphorylated ErbB3, ErbB2, EGFR, MuSK, and AXL were quantified using the ImageJ program (B). (C, D) Assessment of RTK activation in ectopic lesions of mice with endometriosis treated with PCB126 (1 mg/kg) or vehicle once weekly for 5 weeks (n = 3 per group) (C). Phosphorylated AXL and ErbB2 levels were quantified using ImageJ (D). (E, F) Evaluation of the effect of the AXL inhibitor BMS-777607 on endometriosis progression. PCB126-treated mice with endometriosis (n = 4 per group) were treated with BMS-777607 (25 mg/kg) or vehicle 5 times per week for 3 weeks (E). The volumes of ectopic lesions from treated mice were quantified (F). * *P* < .05; ** *P* < .01; *** *P* < .001. Abbreviations: Eut, eutopic endometrium; Ect, ectopic lesions; NS, nonspecific; RTK, receptor tyrosine kinase.

How does PCB126 activate ESR2 in endometriotic lesions to promote endometriosis progression? RTK signaling has been shown to modulate ESR2 activity (35, 36), and PCB126 is known to influence RTK signaling pathways involved in regulating various cellular processes (37, 38). RTK activation contributes to endometriosis progression and is considered a potential molecular therapeutic target for its treatment (39). These observations suggest that PCB126 enhances ESR2 activation by RTK signaling. To test this hypothesis, a mouse RTK array was performed using control endometrial tissue from healthy mice, as well as eutopic and ectopic endometria from mice with endometriosis (Fig. 3A). The levels of phosphorylated ErbB2, EGFR, MuSK, and Axl were markedly elevated in ectopic lesions compared to both eutopic and control endometria (Fig. 3B). These findings indicate that the activation of ErbB2, EGFR, MuSK, and Axl is associated with endometriosis progression in mice. The next question was which RTK(s) are specifically involved in PCB126-induced endometriosis progression. To address this, a mouse RTK array was conducted on ectopic lesions isolated from mice with endometriosis treated with either PCB126 or vehicle (Fig. 3C). PCB126 exposure significantly increased the levels of phosphorylated Axl and phosphorylated ErbB2 in endometriotic lesions (Fig. 3D). Among the RTKs associated with endometriosis, Axl and ErbB2 were preferentially activated by PCB126, suggesting their involvement in PCB126-mediated disease progression. Given that Axl has been more extensively studied in the context of endometriosis, we focused on its activation by PCB126 for further investigation (40, 41).

Although Axl activation is associated with PCB126-induced endometriosis, direct evidence demonstrating its causal role in disease progression is lacking. To address this, we employed the Axl inhibitor BMS-777607, which has been shown to specifically reduce Axl phosphorylation, tumor invasion, and angiogenesis in glioma cells (42). Mice with endometriosis exposed to PCB126 were treated with either BMS-777607 or vehicle as a control. BMS-777607 treatment significantly reduced the volume of endometriotic lesions in PCB126-exposed mice compared to the vehicle-treated group (Fig. 3E and 3F). These results indicate that Axl activation is critical in mediating PCB126-induced endometriosis progression.

**Figure 4.**
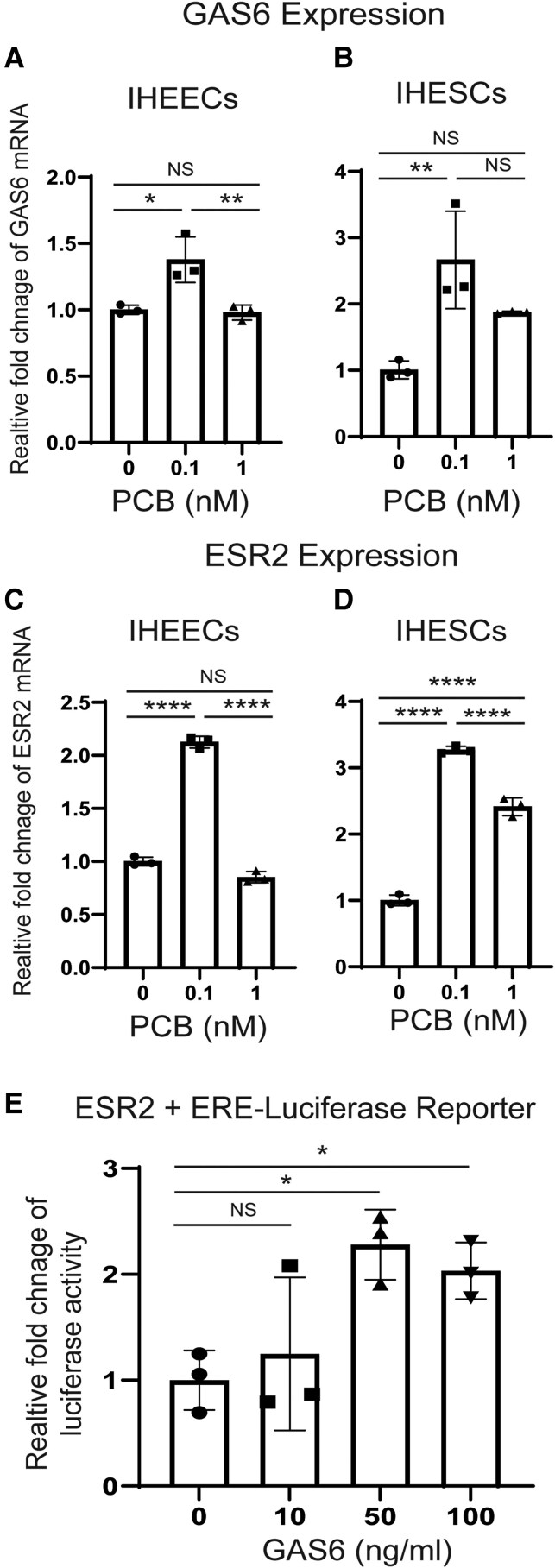
PCB126 elevated levels of GAS6/ESR2 axis to enhance ESR2 activity. (A, B) GAS6 mRNA levels in IHEECs (A) and IHESCs (B) following treatment with various doses of PCB126 or vehicle. (C, D) ESR2 mRNA levels in IHEECs (C) and IHESCs (D) after treatment with increasing doses of PCB126 or vehicle. (E) Assessment of the intrinsic transcriptional activity of ESR2 induced by GAS6. HeLa cells were transiently transfected with an ESR2 expression vector and an ERE-luciferase reporter, followed by treatment with varying concentrations of GAS6 or vehicle for 48 hours. Relative luciferase activity was calculated as the fold change in GAS6-treated vs vehicle-treated cells. * *P* < .05; ** *P* < .01; *** *P* < .001. Abbreviations: ERE, estrogen response element; ESR2, estrogen receptor β; GAS6, growth arrest–specific 6; IHEEC, immortalized human endometrial epithelial cell; IHESC, immortalized human endometrial stromal cell; NS, nonspecific.

The next question is how PCB126 activates Axl in endometriotic lesions. To address this, we measured the mRNA levels of GAS6, the ligand for Axl, in endometrial cells treated with PCB126 compared to vehicle. PCB126 (0.1 nM) significantly increased GAS6 mRNA levels in both IHEECs (Fig. 4A) and IHESCs (Fig. 4B). These results suggest that PCB126 upregulates GAS6 expression, leading to Axl activation in endometriotic lesions. We next asked whether PCB126 also increases ESR2 levels in endometriotic lesions, thereby promoting disease progression. Similar to GAS6, PCB126 (0.1 nM) treatment elevated ESR2 mRNA levels in both IHEECs (Fig. 4C) and IHESCs (Fig. 4D) compared to the vehicle. To further explore whether GAS6 can enhance ESR2 activity, HeLa cells were transiently transfected with an ESR2 expression vector and an ERE-luciferase reporter construct. Treatment with GAS6 (50 ng/mL) significantly increased ESR2 transcriptional activity compared to vehicle-treated controls (Fig. 4E). These findings indicate that, in addition to elevating ESR2 expression, PCB126 enhances ESR2 activity through activation of the Axl/GAS6 signaling axis in endometriotic lesions.

### PCB126 Upregulated Dnmt3a in Endometriotic Lesions

**Figure 5.**
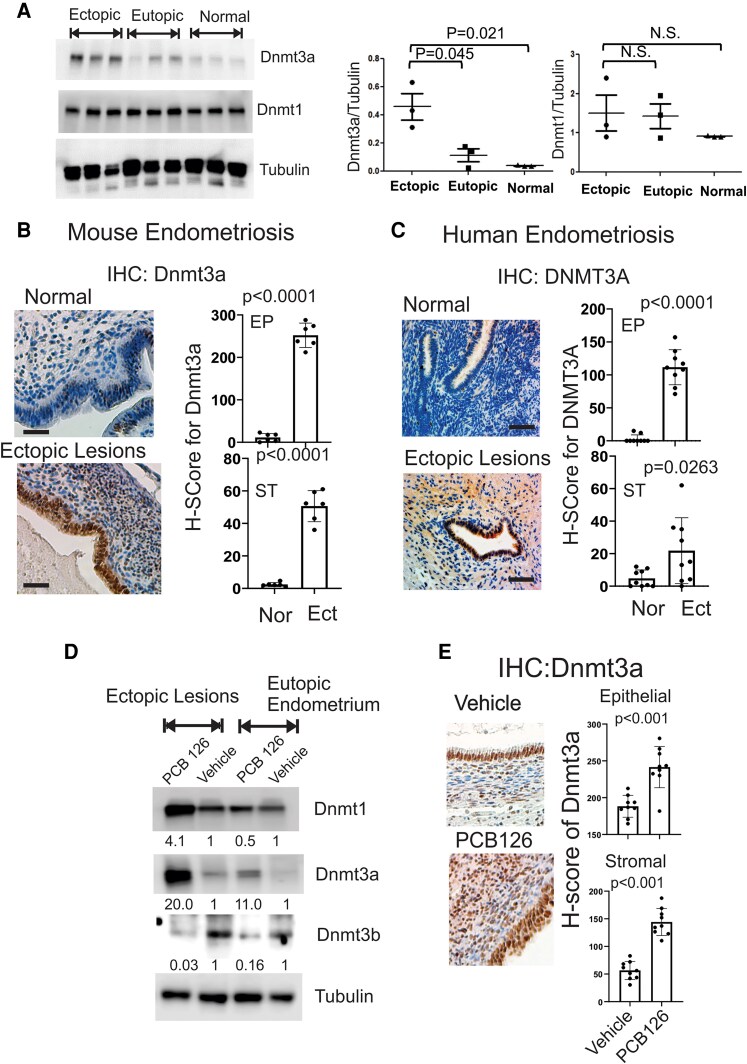
PCB126 increased the level of Dnmt3a in ectopic lesions. (A) Western blot analysis of DNMT3A, DNMT1, and tubulin (as a loading control) in ectopic lesions and eutopic endometrium of mice with endometriosis, as well as in normal endometrium from control mice without endometriosis. The DNMT3A/tubulin and DNMT1/tubulin ratios were quantified for each tissue type. (B) IHC analysis of DNMT3A expression in normal endometrium from control mice and ectopic lesions from mice with endometriosis (n = 6 per group). H-scores were calculated using the program. (C) IHC analysis of DNMT3A expression in normal endometrium from women without endometriosis and ectopic lesions from endometriosis patients (n = 9 per group). H-scores were calculated using the QuPath program. (D) Western blot analysis of DNMT1, DNMT3A, DNMT3B, and tubulin in ectopic lesions and eutopic endometrium from mice with endometriosis treated with PCB126 (1 mg/kg) or vehicle once weekly for 5 weeks. (E) IHC analysis of DNMT3A expression in ectopic lesions from mice with endometriosis treated with PCB126 (1 mg/kg) or vehicle once weekly for 5 weeks. H-scores in epithelial and stromal compartments were quantified using the QuPath program. Scale bar: 50 µm. Abbreviations: DNMT1, DNA methyltransferase 1; DNMT3A, DNA methyltransferase 3A; DNMT3B, DNA methyltransferase 3B; Ect, ectopic lesion; EP, epithelial cells; IHC, immunohistochemistry; Nor, normal endometrium; ST, stromal cells.

Epigenetic dysregulation, such as DNA methylation, is associated with endometriosis progression, and PCB126 exposure has been shown to alter epigenetic states, including DNA methylation (43, 44). However, the functional correlation between PCB126 exposure and endometriosis-associated DNA methylation has not been clearly investigated. To address this question, we investigated whether PCB126 alters the expression of DNMTs in endometriotic lesions compared to control endometrium, potentially promoting endometriosis progression through lesion-specific DNA methylation. Ectopic lesions and eutopic endometrium were isolated from mice with endometriosis and normal uterine tissue from control mice. Western blot analysis showed that Dnmt3a levels were significantly elevated in ectopic lesions compared to both eutopic endometrium and control endometrium (Fig. 5A). In contrast, Dnmt1 levels remained unchanged across ectopic, eutopic, and normal endometrial tissues (Fig. 5A). Therefore, we focused on elucidating the role of Dnmt3a in the progression of endometriosis. IHC analysis further confirmed that Dnmt3a expression was markedly increased in both the epithelial and stromal compartments of ectopic lesions in mice with endometriosis compared to normal endometrium (Fig. 5B). To validate these findings in humans, IHC for Dnmt3a was performed on human endometriotic lesions from endometriosis patients and compared with endometrial tissue from women without the disease. Consistent with the mouse model, Dnmt3a levels were significantly elevated in both epithelial and stromal cells of human endometriotic lesions relative to normal endometrium (Fig. 5C). These results demonstrate that Dnmt3a is upregulated in ectopic lesions in both human patients and mouse models of endometriosis, suggesting a potential role in disease progression.

To determine the effect of PCB126 on Dnmt3a expression in endometriotic lesions, ectopic lesions and eutopic endometrium were isolated from mice with endometriosis treated with PCB126 or vehicle as a control. Western blot analysis showed that PCB126 treatment increased Dnmt1 levels in ectopic lesions but not in eutopic endometrium (Fig. 5D). In contrast, PCB126 significantly elevated Dnmt3a levels in both ectopic lesions and eutopic endometrium compared to vehicle-treated controls (Fig. 5D). Conversely, PCB126 treatment reduced Dnmt3b levels in both ectopic lesions and eutopic endometrium (Fig. 5D). Furthermore, IHC analysis confirmed that PCB126 exposure markedly increased Dnmt3a expression in both epithelial and stromal cells of mouse ectopic lesions compared to vehicle-treated mice with endometriosis (Fig. 5E). These findings indicate that PCB126 exposure significantly upregulates Dnmt3a expression in endometriotic lesions in a mouse model of endometriosis.

### Dnmt3a Is an ERβ Target Gene in Ectopic Lesions

**Figure 6.**
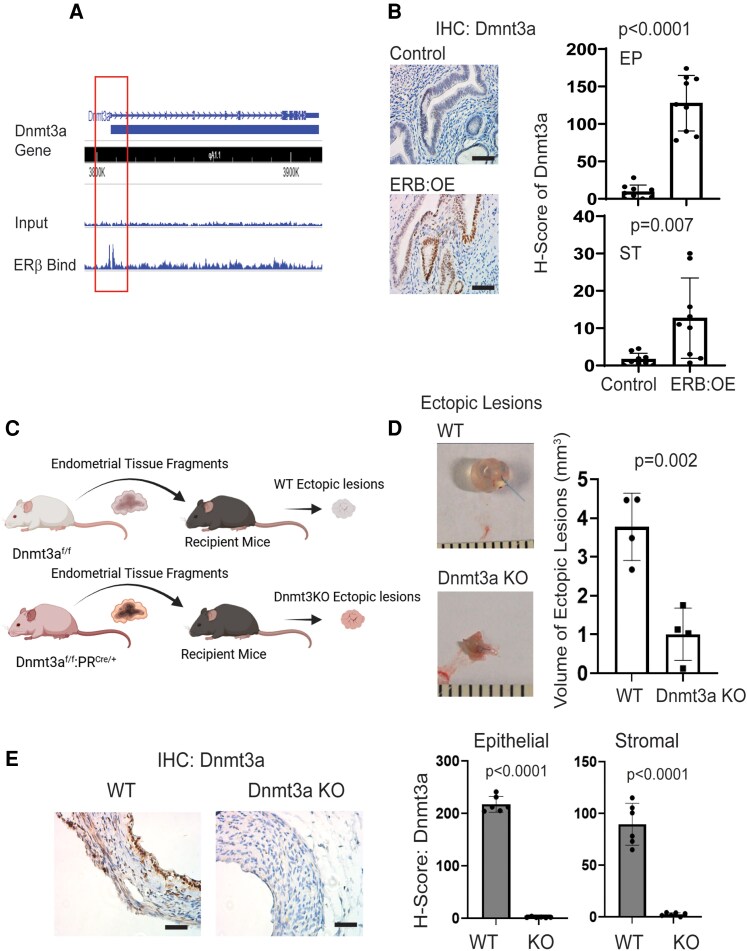
DNMT3A had a critical role in endometriosis progression. (A) ESR2 chromatin immunoprecipitation sequencing analysis revealed direct binding of ESR2 to the promoter region of the *Dnmt3a* gene in ESR2-overexpressing ectopic lesions from mice with endometriosis. (B) IHC using an ESR2 antibody showed that ESR2 overexpression increased DNMT3A levels in the uterus of endometrium-specific ESR2 overexpressing mice compared to control mice. H-scores for DNMT3A in epithelial and stromal cells were quantified using the QuPath program. (C) Schematic overview of the strategy for generating Dnmt3a KO and WT ectopic lesions in syngeneic recipient mice. Endometrial fragments were obtained from the uteri of Dnmt3a^f/f^ :PR^Cre/+^ mice for the KO group and from Dnmt3a^f/f^ mice for the WT control group. (D) Volume analysis of Dnmt3a KO vs WT ectopic lesions. Lesions were harvested from recipient mice on day 21 after endometriosis induction (n = 4 per group). (E) Evaluation of DNMT3A expression in Dnmt3a KO and WT ectopic lesions (from panel D) (n = 4 per group). H-scores for DNMT3A in epithelial and stromal cells of KO and WT lesions were determined using the QuPath program. Scale bar: 50 µm. Abbreviations: DNMT3A, DNA methyltransferase 3A; ESR2, estrogen receptor β; IHC, immunohistochemistry; KO, knockout; PR, progesterone receptor; WT, wild-type.

How is Dnmt3a upregulated in endometriotic lesions? This is a key question for understanding Dnmt3a-mediated endometriosis progression. To investigate this, we reanalyzed a previously published ESR2 chromatin immunoprecipitation sequencing dataset specific to endometriotic lesions (32) and found that ESR2 directly binds to the promoter region of the *Dnmt3a* gene (Fig. 6A). To determine whether ESR2 binding increases Dnmt3a expression in endometriotic lesions, we utilized our ESR2 overexpression mouse model (ROSA^LSL:ESR2/+^:PR^Cre/+^, ERB:OE) in which ESR2 levels are significantly elevated across all compartments of the uterine tissue (20). IHC analysis for Dnmt3a revealed markedly elevated expression in both epithelial and stromal cells of the uterus in ESR2:OE mice compared to control mice (PR^Cre/+^) (Fig. 6B). These results indicate that ESR2 functions as a key transcriptional regulator that directly upregulates Dnmt3a expression in the uterine endometrium. Therefore, PCB126-induced activation of the AXL/GAS6 axis stimulated ESR2, leading to increased Dnmt3a expression in ectopic lesions. This observation may provide a critical clue to fill the knowledge gap in understanding how PCB126 exposure enhances endometriosis progression.

### Dnmt3a Has a Critical Role in Endometriosis Progression

To determine whether Dnmt3a plays an essential role in endometriosis progression, we generated an endometrium-specific Dnmt3a KO mouse by crossing floxed Dnmt3a (Dnmt3a^f/f^) (22) with PR^Cre/+^ mice, in which Cre recombinase is expressed in PR-expressing cells (21). To generate Dnmt3a KO ectopic lesions, uteri were isolated from Dnmt3a^f/f^:PR^Cre/+^ female mice, and endometrial fragments were implanted into syngeneic female recipient mice (Fig. 6C). As a control, uteri were isolated from Dnmt3a ^f/f^ female mice, and control endometrial fragments were similarly implanted into syngeneic recipients (Fig. 6C). Comparative analysis revealed that the volume of Dnmt3a KO ectopic lesions was significantly smaller than that of control lesions (Fig. 6D). To validate Dnmt3a deletion in the ectopic lesions, IHC with a Dnmt3a-specific antibody was performed on both KO and control lesions. The staining confirmed that Dnmt3a expression was absent in both epithelial and stromal cells of Dnmt3a KO lesions, while it was readily detected in control lesions (Fig. 6E). Collectively, these results demonstrate that Dnmt3a plays a critical role in the progression of endometriosis in this mouse model.

### Dnmt3a Regulates Inflammatory Immune Response in Ectopic Lesions for the Progression of Endometriosis

**Figure 7.**
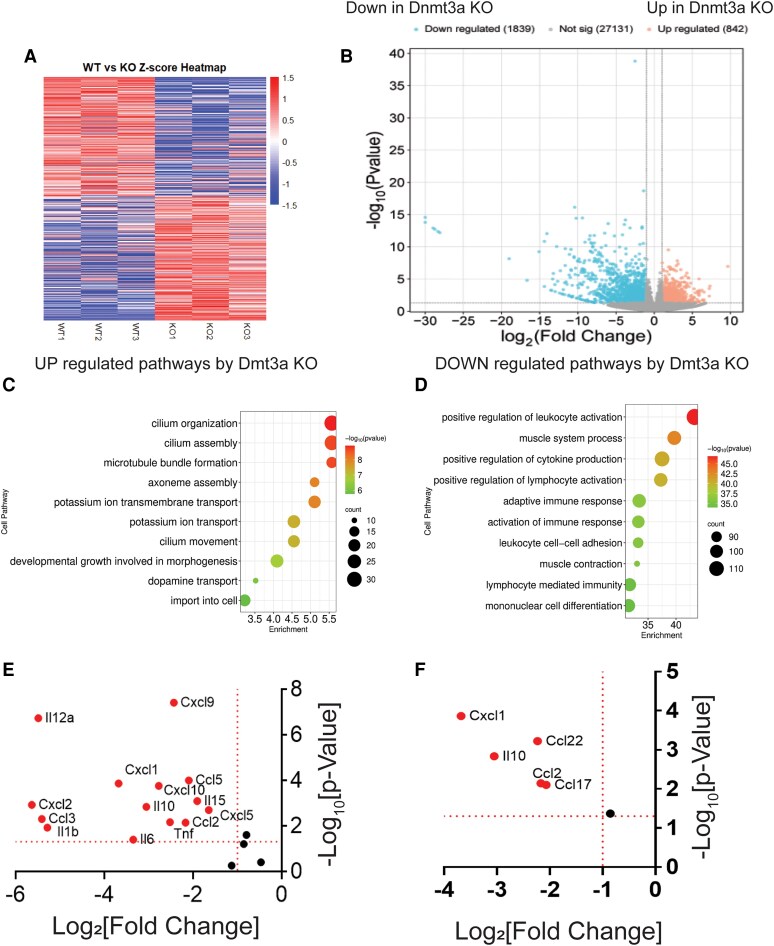
DNMT3A has a critical role in cytokine-mediated immune response in ectopic lesions in mice with endometriosis. (A) Z-score heatmap analysis of Dnmt3a KO (n = 3) and WT (n = 3) ectopic lesions from recipient mice with endometriosis. (B) Volcano plot showing differential gene expression in *Dnmt3a* KO vs WT ectopic lesions. Genes with significant changes (log_2_[fold change] ≥ ± 1, -log_10_[*P*-value] > 1.3) are highlighted in red. (C, D) Gene set enrichment analysis with bubble plots illustrating gene ontology terms enriched in upregulated (C) and downregulated (D) genes in *Dnmt3a* KO ectopic lesions. Analyses were based on normalized enrichment score, false discovery rate, and gene counts. (E) Expression levels of cytokines and chemokines previously implicated in endometriosis progression. (F) Expression levels of cytokines and chemokines known to contribute to an immunosuppressive microenvironment by recruiting regulatory T cells and myeloid-derived suppressor cells. Abbreviations: DNMT3A, DNA methyltransferase 3A; KO, knockout; WT, wild-type.

How does Dnmt3a drive endometriosis progression? To address this key question, RNA expression profiles of control ectopic lesions (n = 3) and *Dnmt3a* KO ectopic lesions (n = 3) were analyzed using bulk RNA sequencing. A heatmap of Z-scores revealed distinct RNA expression patterns between *Dnmt3a* KO and control ectopic lesions (Fig. 7A). Differential gene expression analysis identified 842 genes significantly upregulated in *Dnmt3a* KO ectopic lesions (−log₁₀[*P*-values] > 1.3 and log₂[fold change] > 1.0). In contrast, a substantially larger number of genes (1839) were significantly downregulated in *Dnmt3a* KO lesions compared to controls (−log₁₀[*P*-values] > 1.3 and log₂[fold change] > −1.0) (Fig. 7B). Gene ontology pathway enrichment analysis using the SR plot program (45) revealed significant upregulation of pathways related to cilium organization, assembly, and movement in *Dnmt3a* KO lesions compared to controls (Fig. 7C). In contrast, immune-related pathways, including cytokine production, lymphocyte activation, and adaptive immune responses, were markedly and significantly downregulated in *Dnmt3a* KO ectopic lesions relative to control ectopic lesions (Fig. 7D). To validate this observation, we selected cytokines and chemokines known to play critical roles in endometriosis (46-48). Most of these cytokines and chemokines were significantly reduced in Dnmt3a KO ectopic lesions compared to control ectopic lesions (Fig. 7E). These findings suggest that Dnmt3a is a key driver of cytokine and chemokine expression associated with endometriosis. Endometriotic lesions establish a profoundly immunosuppressive microenvironment that supports ectopic tissue survival and progression. Consequently, regulatory T cells, myeloid-derived suppressor cells, and M2 macrophages are highly enriched within ectopic lesions (49-51). Based on these observations, we measured the expression levels of *Cxcl1*, *Ccl22*, *Ccl2*, *Ccl17*, and *Il10*, as these chemokines are involved in the recruitment of immunosuppressive cells (52, 53). The levels of these chemokines were significantly reduced in Dnmt3a KO endometriotic lesions compared to control ectopic lesions (Fig. 7F). These results indicate that Dnmt3a plays a critical role in establishing the immunosuppressive microenvironment of ectopic lesions, thereby promoting endometriosis progression.

## Discussion

Exposure to endocrine-disrupting chemicals (EDCs), such as PCBs, TCDD, BPA, and their analogs, is strongly associated with the progression of endometriosis by activating multiple intracellular signaling pathways, including those related to inflammation, estrogen and progesterone signaling, cell survival, and apoptosis (3). In addition to EDCs, estrogen is recognized as a key driver of endometriosis progression (54). Therefore, synergistic interaction between estrogen and EDC signaling is likely critical for the progression of endometriosis. How EDCs coordinate with estrogen signaling in endometriotic lesions to promote disease progression remains unclear. A previous study demonstrated that PCB126 can directly induce transcriptional activation of ESR1 and estrogenic responses in the absence of ER agonists, owing to its intrinsic estrogenic activity (55). However, no direct evidence demonstrates the agonistic activity of PCB126 toward ESR2. To address this key question, we identify PCB126, a widespread, dioxin-like environmental contaminant, as a potent enhancer of endometriotic lesion progression via the AXL/ESR2/DNMT3A signaling cascade.

The 1 mg/kg dose of PCB126 was selected based on established toxicological protocol shown to activate AhR and induce epigenetic response in murine models (56, 57). In typical humans, exposure to PCBs is in the low μg/kg range or less (58). Although this exceeds average environmental exposure levels observed in the general population, it reflects acute or high cumulative exposure scenarios relevant to individuals residing near contaminated industrial sites or those with occupational contact, such as in electronic waste or transformer-related industries (59). For example, PCB-exposed electrical workers found blood PCB levels of 88 to 1 319μg/kg after long-term occupational exposure (59).

How does PCB126 activate ESR2 in endometriotic lesions to promote disease progression? This study demonstrates that PCB126 enhances ESR2 activity by activating AXL receptor tyrosine kinase. AXL activation is closely associated with endometriosis progression. For instance, the expression of *GAS6*, the ligand for AXL, and *AXL* mRNA levels are significantly elevated in endometriotic endometrial tissue compared to normal endometrium (40). Therefore, the GAS6–AXL signaling pathway is believed to be aberrantly activated in endometriotic lesions, contributing to disease progression by enhancing ESR2 activity, as GAS6 has been shown to stimulate the intrinsic transcriptional activity of ESR2. The ability of PCB126 to enhance ESR2 activity and stimulate endometriosis lesion growth via AXL represents a unique estrogen mimicry mechanism by which persistent organic pollutants promote estrogen-sensitive pathologies. The next critical question is how the AXL/GAS6 axis activates ESR2 in endometriotic lesions. Unfortunately, there is currently no direct evidence addressing this mechanism. Notably, activation of the AXL/GAS6 axis initiates several downstream signaling pathways, including PI3K–AKT–mTOR, MEK–ERK, NF-κB, and JAK/STAT (60). Activation of these kinase signaling pathways is strongly associated with endometriosis progression (61-64). Moreover, these pathways have been shown to enhance the transcriptional activity of ESR2 (65, 66) Therefore, we propose that kinases activated by the PCB126/AXL/GAS6 axis may, in turn, activate ESR2 to promote endometriosis progression. Further studies are needed to identify which AXL downstream kinase specifically activates ESR2 in endometriotic lesions.

Alterations in epigenetic regulation are strongly associated with the progression of endometriosis (43). In patients with endometriosis, DNMT1, DNMT3A, and DNMT3B are overexpressed in the epithelial component of endometriotic implants compared to normal controls or the eutopic endometrium of women with endometriosis (17, 43). However, other studies have reported significantly lower expression levels of DNMTs in endometriotic lesions compared to the eutopic endometrium of women with endometriosis and to disease-free controls (18). There are controversial studies regarding the differential expression of DNMTs in endometriotic lesions compared to normal endometrium. Therefore, the expression profile of DNMTs in endometriosis may be context-dependent. Our study revealed that DNMT3A levels are elevated in both mice and human endometriotic lesions. Identifying the causal factor responsible for DNMT3A upregulation in endometriotic lesions is a key question for understanding the molecular etiology of endometriosis progression. However, this question remains unresolved. Here, we propose that exposure to PCB126 drives the upregulation of DNMT3A, leading to the establishment of endometriosis-associated DNA methylation patterns that promote disease progression. Notably, PCB126 exposure alters global DNA methylation levels in a context-dependent manner. For example, in elderly Swedish individuals, elevated levels of PCB126 were associated with global DNA hypermethylation (67). However, some studies have linked PCB exposure to global DNA hypomethylation (68). These global methylation changes could be linked to the dysregulation of DNMT expression induced by PCB126. For example, PCB exposure significantly increased DNMT3A and DNMT3B expression in the Leydig cells of progeny rats, impairing testosterone production (69). In contrast, a mixture of PCBs—including PCB126—reduced the expression of DNMT1, DNMT3A, and DNMT3B in the livers of female offspring (44). Therefore, PCB exposure alters DNMT levels in a context-dependent manner. Our study demonstrated that PCB126 elevates DNMT3A expression in endometriotic lesions, contributing to establishing endometriosis-associated DNA methylation.

How is DNMT3A upregulated in endometriotic lesions? Answering this question is critical to understanding DNMT3A-mediated endometriosis progression. Activating protein 2 α and octamer-binding transcription factor 1 have been shown to transactivate DNMT3A expression (70, 71). However, EndometDB analysis revealed that these transcription factors are not upregulated in endometriotic lesions compared to normal endometrium (72). Our study identified ESR2 as a causal factor that enhances DNMT3A expression in endometriotic lesions. ESR2 is a well-established driver of endometriosis, and PCB126 enhances ESR2 activity through the AXL/GAS6 signaling axis. Therefore, the PCB126/AXL/GAS6/ESR2 axis represents a key mechanism underlying the induction of endometriosis-associated DNA methylation via DNMT3A. This provides an important insight into how PCB126 exposure alters DNA methylation to promote endometriosis progression.

Although elevated DNMT3A levels are associated with endometriosis progression, no direct evidence demonstrates that alterations in DNMT3A directly drive disease progression. In this context, our endometrium-specific Dnmt3a knockout mouse model provides critical insight, showing that DNMT3A plays a causal role in endometriosis progression. This raises the question: what is the functional role of DNMT3A in this process? A previous study reported that 15.4% of the variation in endometriosis is attributable to DNA methylation and identified significant alterations in DNA methylation profiles associated with stage III/IV disease. These changes were linked to dysregulation of cellular proliferation, extracellular matrix-cell interactions, and cancer-associated signaling pathways (73). In addition to endometriosis-associated cellular pathways, immune dysregulation driven by cytokine imbalance is also a key contributor to disease progression (74). However, the mechanisms underlying immune dysregulation in endometriosis patients remain unclear. Our data suggest that upregulation of DNMT3A by PCB126-ESR2 results in epigenetic remodeling that drives aberrant expression of immune-modulating cytokine contributing to a proinflammatory, endometriotic lesion-promoting environment. This finding provides an important clue for understanding how immune responses are disrupted during endometriosis progression. Interestingly, pathways related to cilium organization and movement were significantly upregulated in *Dnmt3a* KO ectopic lesions compared to controls. Elevated expression of cilium-related genes (eg, *IFT88*, *CEP164*) enhances ciliogenesis, which promotes epithelial cell redifferentiation and suppresses epithelial-to-mesenchymal transition (EMT) (75). Since EMT plays a critical role in endometriosis progression, the elevation of ciliogenesis resulting from *Dnmt3a* loss may suppress disease progression by inhibiting the EMT process. Therefore, *Dnmt3a* represents a promising molecular target for the development of nonhormonal therapies aimed at correcting immune dysregulation in patients with endometriosis.

**Figure 8.**
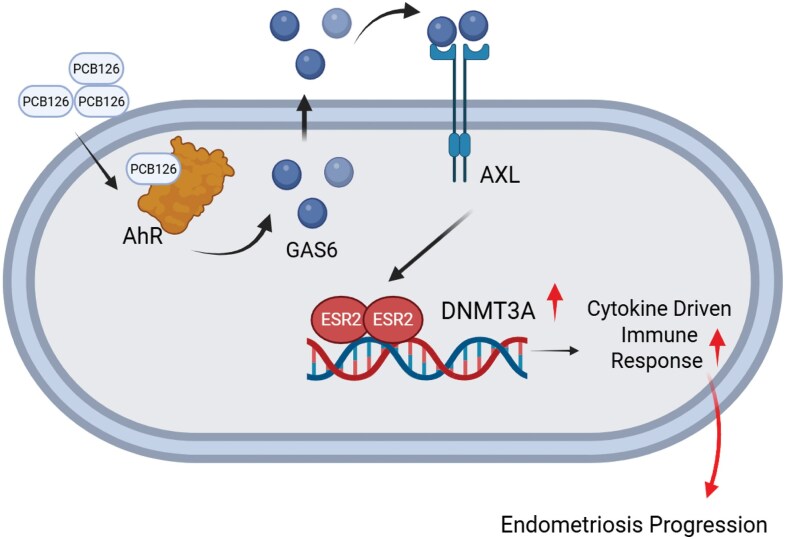
PCB126 indirectly elevated DNMT3A through the AXL/GAS6/ESR2 axis, enhancing endometriosis progression by altering the immune response. PCB126 exposure elevated GAS6 levels in endometriotic lesions, leading to the activation of AXL signaling. Activated AXL enhanced ESR2 activity, which in turn upregulated DNMT3A expression. Elevated DNMT3A contributed to developing endometriosis-associated inflammatory and immune microenvironment, promoting disease progression. Abbreviations: DNMT3A, DNA methyltransferase 3A; ESR2, estrogen receptor β; GAS6, growth arrest–specific 6.

Collectively, our study demonstrated that exposure to PCB126 indirectly upregulated Dnmt3a through the activation of the AXL/GAS6/ESR2 axis, leading to dysregulation of epigenomic regulation in the endometrium and promoting the progression of endometriosis. These findings not only define a novel molecular mechanism by which PCB126 promotes endometriosis (Fig. 8) but also raise important comments about the health risks of persistent organic pollutant hormonally responsive tissues. Targeting the AXL/ESR2/DNMT3A axis may represent a novel therapeutic avenue for environmentally induced endometriosis.

## Data Availability

The datasets used and/or analyzed during the current study are available from the corresponding author on reasonable request. RNA-seq data have been deposited in the Gene Expression Omnibus (GEO) under accession number GSE296259.

## References

[1] Dantkale KS, Agrawal M. A comprehensive review of the diagnostic landscape of endometriosis: assessing tools, uncovering strengths, and acknowledging limitations. Cureus. 2024;16(3):e56978. 38665720 10.7759/cureus.56978 PMC11045176

[2] Sourial S, Tempest N, Hapangama DK. Theories on the pathogenesis of endometriosis. Int J Reprod Med. 2014;2014:179515. 25763392 10.1155/2014/179515 PMC4334056

[3] Dutta S, Banu SK, Arosh JA. Endocrine disruptors and endometriosis. Reprod Toxicol. 2023;115:56‐73. 36436816 10.1016/j.reprotox.2022.11.007

[4] Hoffman CS, Small CM, Blanck HM, Tolbert P, Rubin C, Marcus M. Endometriosis among women exposed to polybrominated biphenyls. Ann Epidemiol. 2007;17(7):503‐510. 17448678 10.1016/j.annepidem.2006.11.005 PMC2075471

[5] Porpora MG, Ingelido AM, di Domenico A, et al Increased levels of polychlorobiphenyls in Italian women with endometriosis. Chemosphere. 2006;63(8):1361‐1367. 16289286 10.1016/j.chemosphere.2005.09.022

[6] Bruner-Tran KL, Osteen KG. Dioxin-like PCBs and endometriosis. Syst Biol Reprod Med. 2010;56(2):132‐146. 20377312 10.3109/19396360903381023 PMC2867352

[7] Louis GM, Weiner JM, Whitcomb BW, et al Environmental PCB exposure and risk of endometriosis. Human Reproduction (Oxford, England). 2005;20(1):279‐285. 15513976 10.1093/humrep/deh575

[8] Martinez-Zamora MA, Mattioli L, Parera J, et al Increased levels of dioxin-like substances in adipose tissue in patients with deep infiltrating endometriosis. Human Reproduction (Oxford, England). 2015;30(5):1059‐1068. 25743783 10.1093/humrep/dev026

[9] Budnik LT, Wegner R, Rogall U, Baur X. Accidental exposure to polychlorinated biphenyls (PCB) in waste cargo after heavy seas. Global waste transport as a source of PCB exposure. Int Arch Occup Environ Health. 2014;87(2):125‐135. 23292295 10.1007/s00420-012-0841-x

[10] Zhang W, Sargis RM, Volden PA, Carmean CM, Sun XJ, Brady MJ. PCB 126 and other dioxin-like PCBs specifically suppress hepatic PEPCK expression via the aryl hydrocarbon receptor. PLoS One. 2012;7(5):e37103. 22615911 10.1371/journal.pone.0037103 PMC3353882

[11] Sechman A, Batoryna M, Antos PA, Hrabia A. Effects of PCB 126 and PCB 153 on secretion of steroid hormones and mRNA expression of steroidogenic genes (STAR, HSD3B, CYP19A1) and estrogen receptors (ERalpha, ERbeta) in prehierarchical chicken ovarian follicles. Toxicol Lett. 2016;264:29‐37. 27832956 10.1016/j.toxlet.2016.11.001

[12] Attar E, Bulun SE. Aromatase and other steroidogenic genes in endometriosis: translational aspects. Hum Reprod Update. 2006;12(1):49‐56. 16123052 10.1093/humupd/dmi034

[13] Mortensen AS, Arukwe A. Estrogenic effect of dioxin-like aryl hydrocarbon receptor (AhR) agonist (PCB congener 126) in Salmon hepatocytes. Mar Environ Res. 2008;66(1):119‐120. 18378297 10.1016/j.marenvres.2008.02.041

[14] Gjernes MH, Schlenk D, Arukwe A. Estrogen receptor-hijacking by dioxin-like 3,3′4,4′,5-pentachlorobiphenyl (PCB126) in Salmon hepatocytes involves both receptor activation and receptor protein stability. Aquat Toxicol. 2012;124-125:197‐208. 22982498 10.1016/j.aquatox.2012.08.015

[15] Rashid CS, Preston JD, Ngo Tenlep SY, et al PCB126 exposure during pregnancy alters maternal and fetal gene expression. Reprod Toxicol. 2023;119:108385. 37080397 10.1016/j.reprotox.2023.108385 PMC10358324

[16] Aluru N, Karchner SI, Glazer L. Early life exposure to low levels of AHR agonist PCB126 (3,3′,4,4′,5-pentachlorobiphenyl) reprograms gene expression in adult brain. Toxicol Sci. 2017;160(2):386‐397. 28973690 10.1093/toxsci/kfx192 PMC5837202

[17] Wu Y, Strawn E, Basir Z, Halverson G, Guo S-W. Aberrant expression of deoxyribonucleic acid methyltransferases DNMT1, DNMT3A, and DNMT3B in women with endometriosis. Fertil Steril. 2007;87(1):24‐32. 17081533 10.1016/j.fertnstert.2006.05.077

[18] Koukoura O, Sifakis S, Spandidos DA. DNA methylation in endometriosis (review). Mol Med Rep. 2016;13(4):2939‐2948. 26934855 10.3892/mmr.2016.4925 PMC4805102

[19] Faul F, Erdfelder E, Lang A-G, Buchner A. G*power 3: a flexible statistical power analysis program for the social, behavioral, and biomedical sciences. Behav Res Methods. 2007;39(2):175‐191. 17695343 10.3758/bf03193146

[20] Han SJ, Jung SY, Wu SP, et al Estrogen receptor β modulates apoptosis complexes and the inflammasome to drive the pathogenesis of endometriosis. Cell. 2015;163(4):960‐974. 26544941 10.1016/j.cell.2015.10.034 PMC4640214

[21] Soyal SM, Mukherjee A, Lee KY, et al Cre-mediated recombination in cell lineages that express the progesterone receptor. Genesis. 2005;41(2):58‐66. 15682389 10.1002/gene.20098

[22] Lavery LA, Ure K, Wan Y-W, et al Losing Dnmt3a dependent methylation in inhibitory neurons impairs neural function by a mechanism impacting Rett syndrome. eLife. 2020;9:e52981. 32159514 10.7554/eLife.52981 PMC7065908

[23] Krikun G, Mor G, Alvero A, et al A novel immortalized human endometrial stromal cell line with normal progestational response. Endocrinology. 2004;145(5):2291‐2296. 14726435 10.1210/en.2003-1606

[24] Bono Y, Kyo S, Takakura M, et al Creation of immortalised epithelial cells from ovarian endometrioma. Br J Cancer. 2012;106(6):1205‐1213. 22353808 10.1038/bjc.2012.26 PMC3304406

[25] Han SJ, Hawkins SM, Begum K, et al A new isoform of steroid receptor coactivator-1 is crucial for pathogenic progression of endometriosis. Nat Med. 2012;18(7):1102‐1111. 22660634 10.1038/nm.2826 PMC3541027

[26] Galaxy Community . The galaxy platform for accessible, reproducible, and collaborative data analyses: 2024 update. Nucleic Acids Res. 2024;52(W1):W83‐W94. 38769056 10.1093/nar/gkae410 PMC11223835

[27] Abolghasemi M, Esmaeilzadeh S, Mirabi P, Golsorkhtabaramiri M. Human exposure to polychlorinated biphenyls (PCBs) and the risk of endometriosis: a systematic review and meta-analysis protocol. Int J Prev Med. 2021;12(1):108. 34760119 10.4103/ijpvm.IJPVM_178_19 PMC8551793

[28] Bankhead P, Loughrey MB, Fernández JA, et al Qupath: open source software for digital pathology image analysis. Sci Rep. 2017;7(1):16878. 29203879 10.1038/s41598-017-17204-5 PMC5715110

[29] Suginami K, Sato Y, Horie A, et al Platelets are a possible regulator of human endometrial re-epithelialization during menstruation. Am J Reprod Immunol. 2017;77(1):1‐8. 10.1111/aji.12609 27868276

[30] Broccardo CJ, Billings RE, Andersen ME, Hanneman WH. Probing the control elements of the CYP1A1 switching module in H4IIE hepatoma cells. Toxicol Sci. 2005;88(1):82‐94. 16081525 10.1093/toxsci/kfi271

[31] Burns KA, Thomas SY, Hamilton KJ, Young SL, Cook DN, Korach KS. Early endometriosis in females is directed by immune-mediated estrogen receptor α and IL-6 cross-talk. Endocrinology. 2018;159(1):103‐118. 28927243 10.1210/en.2017-00562 PMC5761597

[32] Han SJ, Lee JE, Cho YJ, Park MJ, O’Malley BW. Genomic function of estrogen receptor β in endometriosis. Endocrinology. 2019;160(11):2495‐2516. 31504401 10.1210/en.2019-00442 PMC6773435

[33] Trukhacheva E, Lin Z, Reierstad S, Cheng YH, Milad M, Bulun SE. Estrogen receptor (ER) beta regulates ERalpha expression in stromal cells derived from ovarian endometriosis. J Clin Endocrinol Metab. 2009;94(2):615‐622. 19001520 10.1210/jc.2008-1466 PMC2646522

[34] Hall JM, McDonnell DP. The estrogen receptor β-isoform (ERβ) of the human estrogen receptor modulates ERα transcriptional activity and is a key regulator of the cellular response to estrogens and antiestrogens. Endocrinology. 1999;140(12):5566‐5578. 10579320 10.1210/endo.140.12.7179

[35] St-Laurent V, Sanchez M, Charbonneau C, Tremblay A. Selective hormone-dependent repression of estrogen receptor beta by a p38-activated ErbB2/ErbB3 pathway. J Steroid Biochem Mol Biol. 2005;94(1-3):23‐37. 15862947 10.1016/j.jsbmb.2005.02.001

[36] Sugiura H, Miki Y, Iwabuchi E, et al Estrogen receptor β is involved in acquired resistance to EGFR-tyrosine kinase inhibitors in lung cancer. Anticancer Res. 2021;41(5):2371‐2381. 33952462 10.21873/anticanres.15012

[37] Gourronc FA, Helm BK, Robertson LW, et al Transcriptome sequencing of 3,3′,4,4′,5-pentachlorobiphenyl (PCB126)-treated human preadipocytes demonstrates progressive changes in pathways associated with inflammation and diabetes. Toxicol In Vitro. 2022;83:105396. 35618242 10.1016/j.tiv.2022.105396 PMC9423686

[38] Shimada ALB, Cruz WS, Loiola RA, et al Absorption of PCB126 by upper airways impairs G protein-coupled receptor-mediated immune response. Sci Rep. 2015;5(1):14917. 26449762 10.1038/srep14917 PMC4598834

[39] Greenhill C . Targeting endometriosis. Nat Rev Endocrinol. 2024;20(4):193‐193. 10.1038/s41574-024-00959-z 38308073

[40] Sun WS, Misao R, Iwagaki S, Fujimoto J, Tamaya T. Coexpression of growth arrest-specific gene 6 and receptor tyrosine kinases, Axl and sky, in human uterine endometrium and ovarian endometriosis. Mol Hum Reprod. 2002;8(6):552‐558. 12029073 10.1093/molehr/8.6.552

[41] Wu R, Zhou W, Chen S, et al Lipoxin A4 suppresses the development of endometriosis in an ALX receptor-dependent manner via the p38 MAPK pathway. Br J Pharmacol. 2014;171(21):4927‐4940. 24923883 10.1111/bph.12816 PMC4294115

[42] Onken J, Torka R, Korsing S, et al Inhibiting receptor tyrosine kinase AXL with small molecule inhibitor BMS-777607 reduces glioblastoma growth, migration, and invasion in vitro and in vivo. Oncotarget. 2016;7(9):9876‐9889. 26848524 10.18632/oncotarget.7130 PMC4891090

[43] Marquardt RM, Tran DN, Lessey BA, Rahman MS, Jeong J-W. Epigenetic dysregulation in endometriosis: implications for pathophysiology and therapeutics. Endocr Rev. 2023;44(6):1074‐1095. 37409951 10.1210/endrev/bnad020 PMC10638603

[44] Aluru N, Engelhardt J. Exposure to 3,3′,4,4′,5-pentachlorobiphenyl (PCB126) causes widespread DNA hypomethylation in adult zebrafish testis. Toxicol Sci. 2022;188(1):75‐87. 35477799 10.1093/toxsci/kfac044 PMC9237993

[45] Tang D, Chen M, Huang X, et al SRplot: a free online platform for data visualization and graphing. PLoS One. 2023;18(11):e0294236. 37943830 10.1371/journal.pone.0294236 PMC10635526

[46] Oală IE, Mitranovici MI, Chiorean DM, et al Endometriosis and the role of pro-inflammatory and anti-inflammatory cytokines in pathophysiology: a narrative review of the literature. Diagnostics (Basel). 2024;14(3):312. 38337827 10.3390/diagnostics14030312 PMC10855755

[47] Zhou WJ, Yang HL, Shao J, et al Anti-inflammatory cytokines in endometriosis. Cell Mol Life Sci. 2019;76(11):2111‐2132. 30826860 10.1007/s00018-019-03056-x PMC11105498

[48] Borrelli GM, Abrão MS, Mechsner S. Can chemokines be used as biomarkers for endometriosis? A systematic review. Hum Reprod. 2014;29(2):253‐266. 24287816 10.1093/humrep/det401

[49] Li MQ, Wang Y, Chang KK, et al CD4+Foxp3+ regulatory T cell differentiation mediated by endometrial stromal cell-derived TECK promotes the growth and invasion of endometriotic lesions. Cell Death Dis. 2014;5(10):e1436‐e1436. 25275597 10.1038/cddis.2014.414 PMC4649519

[50] Zhang W, Li K, Jian A, Zhang G, Zhang X. Prospects for potential therapy targeting immune-associated factors in endometriosis (review). Mol Med Rep. 2025;31(3):57. 39717957 10.3892/mmr.2024.13422 PMC11715623

[51] Zhang T, Zhou J, Man GCW, et al MDSCs drive the process of endometriosis by enhancing angiogenesis and are a new potential therapeutic target. Eur J Immunol. 2018;48(6):1059‐1073. 29460338 10.1002/eji.201747417 PMC6273458

[52] Ozga AJ, Chow MT, Luster AD. Chemokines and the immune response to cancer. Immunity. 2021;54(5):859‐874. 33838745 10.1016/j.immuni.2021.01.012 PMC8434759

[53] Yi M, Li T, Niu M, et al Targeting cytokine and chemokine signaling pathways for cancer therapy. Signal Transduct Target Ther. 2024;9(1):176. 39034318 10.1038/s41392-024-01868-3 PMC11275440

[54] Bulun SE, Yilmaz BD, Sison C, et al Endometriosis. Endocr Rev. 2019;40(4):1048‐1079. 30994890 10.1210/er.2018-00242 PMC6693056

[55] Mortensen AS, Arukwe A. Activation of estrogen receptor signaling by the dioxin-like aryl hydrocarbon receptor agonist, 3,3′,4,4′,5-pentachlorobiphenyl (PCB126) in Salmon in vitro system. Toxicol Appl Pharmacol. 2008;227(2):313‐324. 18155262 10.1016/j.taap.2007.11.003

[56] Klenov V, Flor S, Ganesan S, et al The aryl hydrocarbon receptor mediates reproductive toxicity of polychlorinated biphenyl congener 126 in rats. Toxicol Appl Pharmacol. 2021;426:115639. 34256052 10.1016/j.taap.2021.115639 PMC8500329

[57] Petri BJ, Piell KM, Wahlang B, et al Polychlorinated biphenyls alter hepatic m6A mRNA methylation in a mouse model of environmental liver disease. Environ Res. 2023;216(Pt 3):114686. 36341798 10.1016/j.envres.2022.114686 PMC10120843

[58] Agarwal M, Hoffman J, Ngo Tenlep SY, et al Maternal polychlorinated biphenyl 126 (PCB 126) exposure modulates offspring gut microbiota irrespective of diet and exercise. Reprod Toxicol. 2023;118:108384. 37061048 10.1016/j.reprotox.2023.108384 PMC10257154

[59] Maroni M, Colombi A, Cantoni S, Ferioli E, Foa V. Occupational exposure to polychlorinated biphenyls in electrical workers. I. Environmental and blood polychlorinated biphenyls concentrations. Br J Ind Med. 1981;38(1):49‐54. 6781529 10.1136/oem.38.1.49 PMC1008798

[60] Gay CM, Balaji K, Byers LA. Giving AXL the axe: targeting AXL in human malignancy. Br J Cancer. 2017;116(4):415‐423. 28072762 10.1038/bjc.2016.428 PMC5318970

[61] Madanes D, Bilotas MA, Bastón JI, et al PI3K/AKT pathway is altered in the endometriosis patient's endometrium and presents differences according to severity stage. Gynecol Endocrinol. 2020;36(5):436‐440. 31637941 10.1080/09513590.2019.1680627

[62] Velarde MC, Aghajanova L, Nezhat CR, Giudice LC. Increased mitogen-activated protein kinase kinase/extracellularly regulated kinase activity in human endometrial stromal fibroblasts of women with endometriosis reduces 3′,5′-cyclic adenosine 5′-monophosphate inhibition of cyclin D1. Endocrinology. 2009;150(10):4701‐4712. 19589865 10.1210/en.2009-0389 PMC2754675

[63] González-Ramos R, Donnez J, Defrère S, et al Nuclear factor-kappa B is constitutively activated in peritoneal endometriosis. Mol Hum Reprod. 2007;13(7):503‐509. 17483545 10.1093/molehr/gam033

[64] Kotlyar AM, Mamillapalli R, Flores VA, Taylor HS. Tofacitinib alters STAT3 signaling and leads to endometriosis lesion regression. Mol Hum Reprod. 2021;27(4):gaab016. 33693775 10.1093/molehr/gaab016 PMC8454207

[65] Skolariki A, D'Costa J, Little M, Lord S. Role of PI3K/Akt/mTOR pathway in mediating endocrine resistance: concept to clinic. Explor Target Antitumor Ther. 2022;3(2):172‐199. 36046843 10.37349/etat.2022.00078 PMC9400772

[66] Fitzpatrick JL, Mize AL, Wade CB, Harris JA, Shapiro RA, Dorsa DM. Estrogen-mediated neuroprotection against beta-amyloid toxicity requires expression of estrogen receptor alpha or beta and activation of the MAPK pathway. J Neurochem. 2002;82(3):674‐682. 12153491 10.1046/j.1471-4159.2002.01000.x

[67] Lind L, Penell J, Luttropp K, et al Global DNA hypermethylation is associated with high serum levels of persistent organic pollutants in an elderly population. Environ Int. 2013;59:456‐461. 23933504 10.1016/j.envint.2013.07.008

[68] Mitchell MM, Woods R, Chi LH, et al Levels of select PCB and PBDE congeners in human postmortem brain reveal possible environmental involvement in 15q11-q13 duplication autism spectrum disorder. Environ Mol Mutagen. 2012;53(8):589‐598. 22930557 10.1002/em.21722 PMC3739306

[69] Thangavelu SK, Mohan M, Ramachandran I, Jagadeesan A. Lactational polychlorinated biphenyls exposure induces epigenetic alterations in the Leydig cells of progeny rats. Andrologia. 2021;53(9):e14160. 34241921 10.1111/and.14160

[70] Guo W, Chen J, Yang Y, Zhu J, Wu J. Epigenetic programming of Dnmt3a mediated by AP2α is required for granting preadipocyte the ability to differentiate. Cell Death Dis. 2016;7(12):e2496‐e2496. 27906176 10.1038/cddis.2016.378 PMC5261006

[71] Zhao J-Y, Liang L, Gu X, et al DNA methyltransferase DNMT3a contributes to neuropathic pain by repressing Kcna2 in primary afferent neurons. Nat Commun. 2017;8(1):14712. 28270689 10.1038/ncomms14712 PMC5344974

[72] Gabriel M, Fey V, Heinosalo T, et al A relational database to identify differentially expressed genes in the endometrium and endometriosis lesions. Sci Data. 2020;7(1):284. 32859947 10.1038/s41597-020-00623-x PMC7455745

[73] Mortlock S, Houshdaran S, Kosti I, et al Global endometrial DNA methylation analysis reveals insights into mQTL regulation and associated endometriosis disease risk and endometrial function. Commun Biol. 2023;6(1):780. 37587191 10.1038/s42003-023-05070-z PMC10432557

[74] Izumi G, Koga K, Takamura M, et al Involvement of immune cells in the pathogenesis of endometriosis. J Obstet Gynaecol Res. 2018;44(2):191‐198. 29316073 10.1111/jog.13559

[75] Blom JN, Wang X, Lu X, Kim MY, Wang G, Feng Q. Inhibition of intraflagellar transport protein-88 promotes epithelial-to-mesenchymal transition and reduces cardiac remodeling post-myocardial infarction. Eur J Pharmacol. 2022;933:175287. 36150531 10.1016/j.ejphar.2022.175287

